# The Flavonoid Components of *Scutellaria baicalensis*: Biopharmaceutical Properties and their Improvement using Nanoformulation Techniques

**DOI:** 10.2174/1568026623666221128144258

**Published:** 2023-01-26

**Authors:** Jilin Wang, Xiaojiao Feng, Ziwei Li, Yiting Liu, Wenzhuo Yang, Tingen Zhang, Pan Guo, Zhidong Liu, Dongli Qi, Jiaxin Pi

**Affiliations:** 1 State Key Laboratory of Component-based Chinese Medicine, Tianjin University of Traditional Chinese Medicine, Tianjin, 301617, China;; 2 Engineering Research Center of Modern Chinese Medicine Discovery and Preparation Technique, Ministry of Education, Tianjin University of Traditional Chinese Medicine, Tianjin, 301617, China;; 3 Haihe, Laboratory of Modern Chinese Medicine, Tianjin University of Traditional Chinese Medicine, Tianjin, 301617, China

**Keywords:** *Scutellaria baicalensis*, Georgi, Baicalin, Baicalein, Wogonin, Biopharmaceutics, Nanoformulations

## Abstract

*Scutellaria baicalensis* georgi, known as “Huangqin” in its dried root form, is a herb widely used in traditional Chinese medicine for “clearing away heat, removing dampness, purging fire and detoxification”. Baicalin, baicalein, wogonin, and wogonoside are the main flavonoid compounds found in *Scutellaria baicalensis*. Scutellaria baicalensis flavonoid components have the potential to prevent and treat a host of diseases. The components of S. baicalensis have limited clinical application due to their low water solubility, poor permeability, and microbial transformation *in vivo*. Nanopharmaceutical techniques can improve their biopharmaceutical properties, enhance their absorption *in vivo*, and improve their bioavailability. However, due to the limited number of clinical trials, doubts remain about their toxicity and improvements in human absorption as a result of nanoformulations. This review summarizes the latest and most comprehensive information regarding the absorption, distribution, metabolism, and excretion of the *Scutellaria baicalensis* components *in vivo*. We examined the main advantages of nanodrug delivery systems and collected detailed information on the nanosystem delivery of the *Scutellaria baicalensis* components, including nanosuspensions and various lipid-based nanosystems. Lipid-based systems including liposomes, solid lipid nanoparticles, nanoemulsions, and self-micro emulsifying drug delivery systems are introduced in detail. In addition, we make recommendations for related and future research directions. Future research should further examine the absorption mechanisms and metabolic pathways of nanoformulations of the components of *Scutellaria baicalensis in vivo*, and accurately track the *in vivo* behavior of these drug delivery systems to discover the specific reasons for the enhanced bioavailability of nanoformulations of the *scutellaria baicalensis* components. The development of targeted oral administration of intact nanoparticles of *Scutellaria baicalensis* components is an exciting prospect.

## 1 INTRODUCTION

**Fig. (1):**
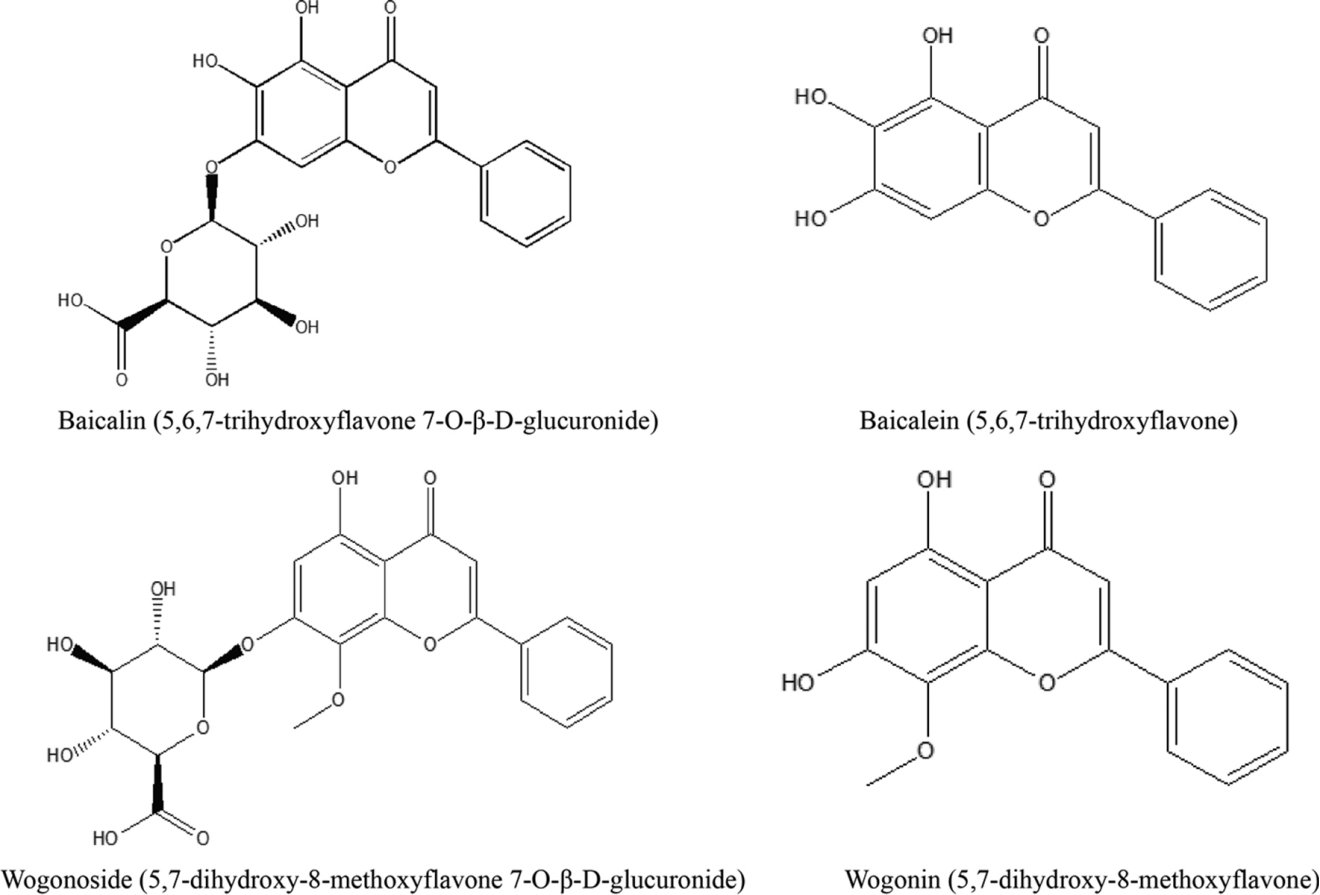
Primary flavonoids, active compounds of *Scutellaria baicalensis.*

*Scutellaria baicalensis* (SB) is a member of the Labiataceae and its dried roots have long been used in traditional Chinese medicine [1]. The active compounds of SB are primarily flavonoids (Fig. **1**), including baicalin (BG, 5,6,7-trihydroxyflavone 7-O-β-D-glucuronide), baicalein (BE, 5,6,7-trihydroxyflavone), wogonoside (WG, 5,7-dihydroxy-8-methoxyflavone 7-O-β-D-glucuronide), and wogonin (WO, 5,7-dihydroxy-8-methoxyflavone) [2]. Pharmacological and clinical studies have demonstrated that SB has the potential to prevent and treat a host of diseases. As an anti-inflammatory, BG inhibits microglia-induced neuroinflammation by inhibiting the activation of NLRP3 inflammatory cells and the TLR4/NF-κB signaling pathway [3]. BE attenuates the pathological changes caused by asthma by inhibiting the NF-κB/iNOS pathway [4]. BG and BE have antitumor properties and inhibit the growth of melanoma cells by inhibiting glucose uptake and metabolism in tumor cells by affecting the mTOR-HIF-1α signaling pathway [5]. BE can inhibit the growth and proliferation of human nasopharyngeal carcinoma cells by changing the cell cycle and inducing apoptosis [6]. WO has antibacterial and antiviral properties and can mediate the effect of herpes simplex virus by regulating the NF-κB and JNK/p38 MAPK pathways [7]. BG can attenuate the inflammatory responses of chicken type II pneumocytes induced by avian pathogenic *Escherichia coli* [8]. Although the SB flavonoid components (SBFC) exhibit some pharmacological activities, their versatility, safety, therapeutic potential, and development as a clinical drug have been severely limited by a series of challenges.

Biopharmaceutical studies often focus on the *in vivo* profiles after drug administration, namely, absorption, distribution, metabolism, and excretion (ADME). The Biopharmaceutics Classification System (BCS) defines drugs according to their solubility and permeability as: class I, high permeability and high solubility; class II, high permeability and low solubility; class III, low permeability and high solubility; and class IV, low permeability and low solubility [9-11]. In contrast to BCS-I drugs, the classes BCS-II, BCS-III, and BCS IV generally suffer from biopharmaceutical problems, including difficulties in dissolving into the body fluid environment or in being absorbed through epithelial cells, resulting in low bioavailability [12]. According to the BCS, the low solubility and permeability of SBFC components such as BG and BE lead to low bioavailability, which limits the clinical application of SBFC [13].

Nanoformulation technology can reduce drugs to nanoscales in different ways to improve drug bioavailability and solubility, reduce systemic side effects, prolong circulation time, and accurately give priority to accumulation in target organs [14]. In addition, nanoparticles can be customized to ensure sustained, controlled release and facilitate drug delivery. Nanoparticles also have the advantage of targeted administration with increased cycle half-life and altered drug configuration due to drug localization and specific absorption in somatic cells [15]. Therefore, nanotechnology can offer particular advantages for BCS-IV drugs, such as BG, which require solubilization and enhanced permeability. This review provides a comprehensive approach to the improvement of the oral absorption of SBFC, especially through the use of nano-based approaches to improve the efficacy of oral administration of SBFC.

## 2 ADME BEHAVIOR OF THE FLAVONOID COMPONENTS OF *SCUTELLARIA BAICALENSIS IN VIVO*

### 2.1 Absorption

While the flavonoid aglycones of SBFC have good membrane permeability, the flavonoid glycosides have poor membrane permeability due to their potentially high number of hydrogen bonds [16].

BE shows favorable permeability and lipophilicity. It is absorbed by passive diffusion in the intestine and then metabolized to BG in the intestinal and liver cells. BG is the main metabolite found in the bloodstream after oral administration of BE or BG [17]. The high polarity of BG restricts simple diffusion across the lipid bilayer, so carrier mediation is required for its absorption *in vivo*. Studies have shown that the multidrug-resistant proteins (MRP) MRP2 and MRP3, and breast cancer resistance protein (BCRP) are basolateral and apical transporters of BG, respectively [17, 18]. The affinities between BG and its transporter ranks: BCRP > MRP3 > MRP2 [19]. The absorption sites of BG in rats were preliminarily evaluated using the *in situ* absorption method in rat stomachs and various intestinal segments. The results demonstrated that there were two sites of BG absorption: the first, in the upper intestine, possibly through direct absorption; and the second, in the colon in the form of aglycon [20]. In MRP2-deficient rats, the area under the curve (AUC) and Cmax of BG were five and eight times higher than in MRP2-competent rats, respectively [21].

To study and compare the absorption sites of BG and BE *in vivo*, both were perfused *in situ* in rats with and without bile duct ligation. The results showed that BG was well absorbed in the stomach but poorly absorbed in the small intestine and colon, while BE was better absorbed in the stomach and small intestine but poorly absorbed in the colon. It also demonstrated that bile could excrete BG and noticeably accelerate the absorption of BE [22]. When BG was orally administered at an equimolar dose of BE, their Tmax max values differed markedly, showing that BG was absorbed more slowly than BE. This suggested that BG may be absorbed only when it is hydrolyzed by enterobacteria in the colon, while BE is absorbed directly through the small intestine [23]. In antibiotic-treated rats, antibiotics decreased the transformation of BG into BE by inhibiting the intestinal microflora, thus affecting the absorption and metabolism of BG [24]. Nanoformulations could improve the bioavailability of both orally administered SBFC and BG nanosuspensions [25] and BG liposome [26].

### 2.2 Distribution

BG was found to accumulate in various tissues. After intravenous administration of BG, its concentration was highest in the kidneys and plasma. Its concentration in the major organs decreased as follows: kidney > lung > spleen [27]. The distribution of BE and WO was similar *in vivo*. In addition to their glucuronides and sulfates, free forms of BE and WO were also present in the tissues of the liver, kidneys, and lungs. BG was the primary component found in the lung, while GLUCURONIDES and sulfate were predominant in the liver and kidney. WO was the main component in the liver, kidney and lung, and a small amount of WO glucuronide and sulfates were detected in the kidney and liver. BE and WO, or their glucuronides and sulfates, were not detected in the brain [28]. The results of intravenous injection were similar to those for oral administration, the levels of WO in the kidney and liver being noticeably higher than in the other tissues. Following intravenous injection of WO, the WO content of testis tissue was close to that of the heart, stomach, and spleen, indicating that WO could effectively cross the blood-testis barrier and that the movement of WO from plasma to tissue was very fast and increased with time [29]. In one study, differences were found in the pharmacokinetics and tissue distribution of SBFC between diabetic and normal rats. In normal rats, SBFC was detected in all assayed tissues and showed a wide and diverse distribution, while in rats with type 2 diabetes these components showed high concentrations in the kidneys and lungs particularly [30].

The studies differed in their reports of the distribution of SBFC in brain tissue. In one WG, WO and BE were detected in the brain, indicating that they passed through the blood-brain barrier (BBB). It is noteworthy that BG was not detected in the brain, although its metabolites were found there [31]. In another study, a relatively low level of SBFC was detected in the brain compared to the level in the plasma, indicating that SBFC does not easily cross the BBB; BE was detected in the brain but not quantified [32]. In contrast, Wang *et al*. (2011) did not identify SBFC in rat brains, even after seven repeated oral administrations of SB decoction. This might be related to the sensitivity of the test method and the instruments used for detection, which could detect SBFC in the brain in another study [32].

Besides, the weak permeability of SBFC across BBB could be potentially improved or modified by some nanofor-mulations such as solid lipid nanoparticles [33] and nanocrystals [34].

### 2.3 Metabolism

**Fig. (2):**
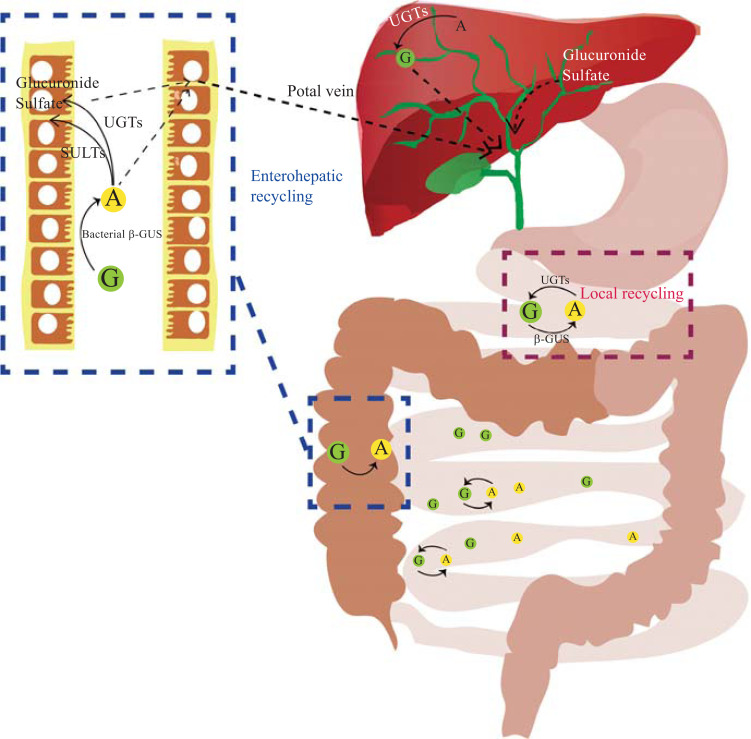
Different recycling schemes applicable to primary flavonoid active compounds of *Scutellaria baicalensis in vivo*. **Abbreviations:** G:glycoside forms of flavones; A:aglycon form of flavones; β-GUS: β-glucuronidase; UGTs: UDP-glucuronosyltransferases; SULT: sulfotransferases.

Regarding SBFC, only aglycones and flavonoid glucosides are absorbed in the small intestine. They are then rapidly metabolized into glucuronidated, methylated, or sulfated metabolites in the jejunum and ileum of the small intestine. After metabolism in the intestine, the flavonoids are further metabolized in the liver by various forms of conjugation reactions, including methylation, sulfation, and glucuronidation [35] (Fig. **2**). The unabsorbed flavonoids are further metabolism by bacterial enzymes in the colon [36].

After glucuronidation, the enterohepatic circulation of BG may include the sequential processes of hepatic uptake from the blood, excretion from the liver to the bile, and transportation of reabsorbed bile from the intestine to the duodenum, before it enters the liver *via* the portal circulation. During this process, there may be both direct absorption in the upper intestine and aglycone absorption in the colon [20, 37]. Bacteria in the gastrointestinal tract could rapidly produce BG disaccharide and isomerize it to form baicalein 6-O-glucuronic acid, which is consistent with the fact that C-6 isomers showed higher systemic exposure than BG [38, 39]. Compared with normal rats, germ-free rat BG showed a lower recovery rate after oral administration and only a small amount of BG was detected in the plasma [24, 40].

As in the intestine, the liver also extensively metabolized SBFC. A total of 32 metabolites were found in rat plasma and urine after oral administration of BG. A number of related reactions have been identified *in vivo*, including methylation, hydroxylation, methoxylation, glucuronide conjugation, sulfate conjugation, and their combined reactions. The results showed that the liver and kidney were important organs for the accumulation of BG metabolites [41]. In another study, several mono- and di- conjugates of BE were found in the bile, mainly glucuronic acid, sulfate, and methylates. The liver plays a significant role in the metabolism of BE and the transport of its conjugated metabolites [42].

*In vitro* studies have shown that the extent of phase II metabolism of BE during transport was dose-dependent. The lipophilic properties of these conjugated metabolites were significantly different from those of their parent compounds due to the polar structure of glucuronic and sulfuric acids, indicating a significant reduction in their ability to cross cell membranes by diffusion. Therefore, the sulfates and glucuronic acid formed by BE in cells were efficiently expelled from both surfaces of the Caco-2 monolayer, and in lesser quantities under the action of MAP inhibitors, indicating the involvement of MRPs in the transport of BE-bound metabolites [17]. Compared with intestinal absorption and deposition of BE and WO, the transport and metabolism of WO resembled that of BE in a Caco-2 cell monolayer model. During bidirectional transport, extensive phase II metabolism of WO has been observed in both glucuronidation and sulfation forms [43].

In rats, WG was detected primarily in the portal vein and body plasma, as well as in the bile. WO was detected only in the small intestine and liver and was rapidly metabolized to WG, which was the only glucuronide metabolite of WO found [44]. This study also demonstrated that OATP2B1 and OATP1B3 mediated the hepatic uptake of WG, and that MRPs mediated the efflux of WG to the bile and circulation. *In vitro* experimental data demonstrated that UGT1A3 and UGT1A7-1A10 were the main families involved in the glucuronidation of WO [45].

### 2.4 Excretion

General data from studies on SBFC demonstrated that most glucuronidates in this molecule could be excreted through the bile, with a little urinary contribution. Bile excretion is the main route of BG excretion and MRP2 is one of the main transporters of bile outflow [21]. The amount of BG and its conjugate metabolites excreted in the urine was negligible compared to bile excretion [21, 37]. Li *et al*. (2011) investigated the hepatic metabolism and disposal of BE by the coupling of binding enzymes and transporters and found that in addition to the intestine, a wide range of METABOLISM also occurs in the liver. Transport studies have shown that, in addition to MRPs and BCRP, human OATP2B1 and OATP1B3 may also mediate the secretion of baicalein-7-Glucuronic acid into the bile in the liver [42]. BE is mainly excreted in bile, with little excretion in urine (0.7%) and about 27.1% as unchanged forms excreted in the feces [46]. WG reached a relatively high level in urine, while only a small amount of aglycones were excreted through urine. On the contrary, aglycones were found at high levels in feces, while the glycoside contents were relatively low. In rats, only about 21% of WO was excreted in its unmodified form in feces, urine, and bile [29, 47].

### 2.5 Nano-based Approaches to Improve the Efficacy of Biopharmaceuticals

Compared with traditional drug delivery systems, nanoparticles have advantages due to their small size and correspondingly large surface area. Although increasing drug solubility is a common method for increasing bioavailability, the delivery of intact nanoparticles through the gastrointestinal mucosa provides another means of significantly improving bioavailability and the use of appropriate nanotechnology to enhance the permeability of epithelial cells or mucosa constitutes a promising strategy. Drug delivery systems containing lipid components, such as liposomes [48], solid lipid nanoparticles [49], nanoemulsions [50], and self-emulsifying systems [51] could improve drug targeting and circumvention of P-glycoprotein efflux from intestinal epithelial cells so improving the absorption of drugs *in vivo*. Nanosuspension delivery systems could reduce particle size and increase the surface area to improve drug solubility in the gastrointestinal tract, thus promoting drug bioavailability [52].

### 2.6 Nanosuspensions

Nanosuspensions are composed of pure drug nanocrystals (100-1,000 nm) dispersed in a liquid medium (usually water) and a minimum amount of stable surfactant [53]. They have shown a number of advantages in the oral use of insoluble drugs, including improved oral absorption, increased bioavailability, rapid onset, reduced variability in eating/fasting state, and reduced variability among subjects [54]. There are two main ways to prepare drug nanosuspensions: top-down and bottom-up. The top-down method involves nanocrystallization and primarily uses mechanical forces applied through high-pressure homogenizers and grinding machines to apply mechanical attrition and reduce the size of large powder particles. Bottom-up approaches involve dissolving a drug in a solvent and subsequently precipitating it by mixing it with a nonsolvent to produce a nanosuspension with the desired particle size distribution. Problems with this method are the production of solvent residues and avoiding crystal growth, and the need for strict process control of the solubility of the drug in at least one solvent [55]. The *in vivo* properties of insoluble drugs could be improved by reducing the particle size of the drug because smaller particles mean an increase in the contact area, which increases the saturation solubility and dissolution rate, resulting in a higher concentration gradient between the intestinal mucosa and the blood to increase absorption and oral bioavailability [56].

The absorption and distribution of SBFC *in vivo* can be effectively improved by preparing its components into a nanosuspension. Nanocrystals prepared using an ultrasonic-homogenization-fluid bed drying process have improved the absorption rate and bioavailability of BG *in vivo*. *In situ* intestinal perfusion experiments showed that BG nanosuspension had obvious advantages over other preparations in terms of dissolution and absorption properties, because improved adhesion could prolong the residence time of the drug on the surface of intestinal bacteria, which helped BG convert to BE and made it easier to absorb [25]. The AUC of novel nanosuspensions with co-processed nanocrystalline cellulose-sodium carboxymethyl starch as a synergetic stabilizer was 2.01-fold higher than that of free BG, which may be due to the decrease in particle size and increase in surface area of BG nanosuspensions, significantly improving the dissolution rate and oral bioavailability [57].

Optimizing the preparation process of BG solid nanocrystals (BG-SNS) was studied using the Box-Behnken design method. Freeze-dried BG-SNS powder had a higher dissolution rate and oral bioavailability compared with both the crude powder and the physical mixture. Under the same conditions, the nanocrystals of the drug had a larger specific surface area, so dissolved more easily in the intestinal fluid. The smaller particles showed better intestinal epithelial cell absorption and permeability than the larger particles [58]. Using nanocrystals modified with a combination of TW80 and TPGS confirmed an approximately 7-fold improvement in the brain exposure of BG compared with the solution [34].

Nanosuspension can also significantly improve the absorption of BE. The NANOCRYSTALS were prepared by antisolvent recrystallization combined with high-pressure homogenization. The average relative bioavailability of NANOCRYSTALS was 1.67 times higher than that of NANOCRYSTALS by oral administration, which had the same pharmacokinetic parameters as those administered intravenously. BE nanocrystals exhibited rapid and extensive absorption. The decrease in particle size and increase in the surface area could lead to increased muco adhesion, which could improve gastrointestinal transit time and lead to improved bioavailability [59]. Subsequently, BE-nicotinamide (BE-NCT) nano-cocrystals with an average particle size of 251.53nm were prepared by high-pressure homogenization method. The integral AUC0-t (6.02times) of oral BE-NCT nano-cocrystals were significantly higher than that of COARSE powder (1 time), BE-NCT cocrystals (2.87 times) and BE nanocrystals (3.32 times), showing excellent performance [60].

### 2.7 Liposomes

Liposomes have a bilayered closed phospholipid vesicular structure and are lipid-based vesicles of spherical shape composed of cholesterol and natural nontoxic phospholipids. Liposomes with particle sizes ranging from 30 nm to a few micrometers can be prepared from natural or synthetic phospholipids and their flexibility is usually regulated by cholesterol in the membrane. Due to their high levels of biocompatibility and low toxicity, and their hydrophilic character, liposomes provide suitable systems for drug delivery. There are many methods of preparing liposomes, including pass-loading techniques and active loading techniques such as mechanical dispersion, solvent dispersion, sonication, and micro-emulsion methods [61-63].

Due to their high biocompatibility, liposomes have been the most widely studied nanodrug delivery system by far and liposome loaded SBFC has been widely studied. Details of these studies are summarized in Table **1**. The use of liposomes improved brain targeting of BG. After administration, the BG concentrations in the heart, liver, spleen, lungs, and brain were all improved but decreased in the kidneys [64]. After intranasal administration, the value of T1/2 and AUC of the BG liposome group was significantly higher than those of the free BG group, leading to higher BG concentrations in brain tissues [26]. BG liposomes were prepared by the effervescent dispersion technique, producing a sustained-release behavior. The results of *in vivo* distribution showed that the drug concentrations of BG liposomes were significantly improved in the liver, kidney, and lung [65]. BG-loaded nanoliposomes had a good therapeutic antitumor effect on nude mice suffering from orthotopic human lung cancer and showed outstanding lung-targeting properties [66]. Glycyrrhetinic acid-modified WO liposomes accumulated rapidly in the liver with a longer retention time and a higher tumor inhibition rate than unmodified WO liposomes [67]. Long-circulating nanoliposomes containing BE showed 4.52 times greater ORAL bioavailability than free BE. Hepatoenteral circulation of BE may appear to be present and the first-pass effect may be avoided [68].

### 2.8 Solid Lipid Nanoparticles

Solid lipid nanoparticles (SLNs) are one of the most promising pharmaceutical colloidal lipid nanocarriers for controlled drug delivery. Their characteristic property is that they are made from solid lipids only, with particle diameters of about 10-1,000 nm. SLNs have the advantages of other carrier systems, including high biocompatibility and bioavailability, controlled release, and protection of incorporated labile drugs from degradation. Furthermore, the lipid degradation products of SLNs, such as glycerides and fatty acids, in the intestinal fluid could promote intestinal transport by producing mixed micelles, thus increasing the absorption of the drug into the intestinal cells [69-71]. There are many ways to prepare SLNs, including high-pressure homogenization, microemulsion, solvent emulsion evaporation, solvent emulsion diffusion, melt diffusion, ultrasonication, double emulsion, membrane contact, and supercritical fluid technology [72].

Only BG delivery has been reported in studies of SBFC in SLNs. The BG-SLNs were prepared using the emulsification/ultrasound method. In pharmacokinetic studies, the AUC value of BG-SLNS was 4.0-fold greater than BG ophthalmic solution, and their C_max_ value was 5.3-fold greater than BG ophthalmic solutions [73]. Subsequently, OX26 antibody conjugation on PEGylated cationic solid lipid nanoparticles (OX26-PEG-CSLN) was prepared using the emulsion evaporation solidification at a low temperature. The results of pharmacokinetic experiments showed that the Cmax of OX26-PEG-CSLN was 7.88 and 1.12 times that of free BG solution and cationic solid lipid nanoparticles (CSLN), respectively, and that the AUC was 11.8 and 1.12 times that of free BG solution and CSLN, respectively [33]. In addition, the effect of OX26-PEG-CSLN on excitotoxic neuronal injury and the pharmacokinetic profile of BG in cerebrospinal fluid during the ischemia-reperfusion period was investigated. The results showed that the AUC and Cmax of OX26-PEG-SNLs were 5.69 and 6.84 times that of free BG solution, respectively. It was further demonstrated that OX26-PEG-SLNs could improve BBB uptake of BG in rats with cerebral ischemia-reperfusion injury [74]. A novel BG-SLNs carrier system was prepared composed of a stearic acid alkaline salt as a lipid substrate using the coacervation method. Contrary to other reports, the BG-SLNs were in a crystalline state rather than an amorphous form due to the adhesion of the nanoparticles. The residence time at the site of absorption was improved and the gastrointestinal transit time was prolonged, because the Cmax and AUC values of BG-SLNs were 1.6-fold and 2.6-fold higher than that of the reference preparation, respectively [75].

### 2.9 Nanoemulsions and SEDDS

Nanoemulsions are composed of two immiscible liquids that disperse drugs as nanodroplets. They are an isotropic, transparent/translucent, heterogeneous system. They can be divided into three types based on the composition of the nanoemulsion: oil-in-water (O/W), water-in-oil (W/O), and dual continuous/multiple emulsion (W/O/W). Two methods were used to prepare the nanoemulsions: a high-energy emulsification method and a low-energy emulsification method. Ultrasonic emulsification, high-pressure homogenization, and membrane emulsification are considered high energy emulsification methods, while the low-energy emulsification method involves temperature phase inversion, emulsion inversion, and spontaneous emulsification [76-78]. As a drug delivery system, nanoemulsion has higher solubilization and better dynamic stability than raw milk. It can improve the therapeutic effect of drugs and reduce adverse reactions and toxicity. Studies have shown that several nanoemulsions were absorbed directly by the lymphatic system, thereby avoiding first-pass metabolism to increase bioavailability [79]. When administered orally, the tiny droplets in the nanoemulsions and their ability to dissolve very hydrophobic drugs provided a way to greatly improve the rate of drug dissolution and subsequent expected systemic bioavailability [80].

Details of these studies are summarized in Table **2**. Due to the sustained-release characteristics of BG-loaded nanoemulsions, they are very effective in improving the oral bioavailability of BG [81]. A novel nanoemulsion improved the systemic exposure of BG. An *in situ* single-pass intestinal perfusion and chylomicron flow-blocking study indicated that intestinal absorption and lymphatic transport were involved in systemic exposure to BG [82]. Nanoemulsions can enhance the concentration of BG in the lymphatic system. An *in vivo* study revealed that a BG nanoemulsion showed increased bioavailability in the delivery of BG to the lymphatic system [50]. Biocompatible nanoemulsions based on hemp oil significantly enhanced the oral bioavailability of BE, with excellent intestinal permeability and transcellular transport ability, and low cytotoxicity [83].

Self-emulsifying drug delivery systems (SEDDS) usually contain one or more hydrophilic cosolvents or emulsifiers, which are isotropic mixtures of drugs, lipids, and surfactants. SEDDS is a broad term that includes droplets with self-emulsifying properties ranging in size from a few nanometers to several microns. The self-micro emulsifying drug delivery system (SMEDDS) is a transparent microemulsion with particles ranging from 100-250 nm. The necessary agitation for self-emulsification is provided by the digestive movements of the stomach and intestines. It is very suitable for lipophilic drugs with limited dissolution rates and could prolong the gastric retention time of drugs to increase their intestinal solubility and permeability [84-86]. Details of these studies are summarized in Table **2**. A preparation for improving oral absorption of BG was developed by combining a phospholipid complex and SMEDDS. The results of Caco-2 cell uptake and single-pass intestinal perfusion models showed that this preparation could significantly improve BG transport and relative bioavailability [87]. The drug release rate and relative bioavailability of SMEDDS were notably higher than that of a free BE suspension [88]. BE SMEDDS with a PHOSPHOLIPID complex as an intermediate not only notably increased the oral bioavailability of BE but also improved the proportion of BE transported by the lymphatic system, which is advantageous in promoting the interaction of BE with the relevant immune cells in the lymphatic system [89].

### 2.10 Other Nanoformulations

There are other nanoscale systems for improving the biopharmaceutical properties of SBFC in addition to those described above. Due to the small number of studies involved, only a brief description is given here, with the details provided in Table **3**.

The mixed micelle system is composed of two or more materials and has better performance than a micelle system comprising only a single carrier material [90]. Mixed micelles have a hydrophobic core that could serve as a reservoir to improve the solubility of water-insoluble drugs [91]. Compared with traditional micelles, mixed micelles could improve drug solubility and stability, and so have the advantages of prolonging circulation time and improving drug bioavailability [92]. A BG-loaded mixed micelle system using Pluronics P123 copolymer and sodium taurocholate as carrier materials had sustained release effects when administered orally. Its intestinal absorption was significantly higher than free BG solution with increased oral bioavailability [93]. BG-polyethylene glycolpoly (lactic-co-glycolic acid) copolymer-loaded nanomicelles had good drug-loading properties, sustained release *in vitro*, and accumulated drugs in the ischemic myocardium, providing excellent cardiac targeting [94].

A new generation of lipid nanoparticles, nanostructured lipid carriers (NLC), are prepared from solid lipids. In contrast to SLNs, NLCs consist of a solid matrix that entraps liquid lipid nanocomposites, effectively preventing problems such as drug loading limitation and drug shedding during storage [95]. BG-PEG-NLC improves the bioavailability of BG, prolongs the retention time *in vivo*, and enhances its efficacy. It provides higher drug concentrations in the heart than free BG solution and could be used as a biocompatible carrier for heart-targeted drug delivery [96]. Free BG modified with the transferrin receptor monoclonal antibody OX26 combined with salvianolic acid B (Sal B) could increase BG content in the brain and enhance brain transmission of SaL B and BG [97]. Tocol NLC loaded with BE could effectively improve the stability of BE and its ability to penetrate the brain [98].

Hydrogel is a kind of swelling polymer network system which uses water as a dispersion medium and has the characteristics of promoting direct contact between the drug and human tissues, allowing long-term adhesion of minimal drug doses to the target tissue [99]. BG-NLC loaded double sensitive hydrogel could prolong the drug residence time on the anterior cornea without causing severe irritation [100]. The dissolution of a drug is directly affected by the dispersibility of the drug in the carrier. Solid dispersion is a new technique for increasing the rate of dissolution of insoluble drugs in solid dispersion, amorphous, and molecular phase carriers (termed simple eutectic mixtures), co-precipitates (also termed glassy solid solutions), and solid solutions, respectively [101]. The pharmacokinetics of BG-polyvinylpyrroli-done co-precipitates prepared by the solid dispersion technique was compared with those of free BG and was found to significantly improve the bioavailability of BG in beagle dogs [102]. A BG-mesoporous carbon solid dispersion prepared by solvent evaporation could significantly shorten the time to T and produce higher Cmax without intestinal and renal toxicity, compared with pure drugs [103].

## CONCLUSION AND PROSPECTS

In this paper, we summarize the ADME behavior of SBFC *in vivo* and the recent progress of ADME improvements using nanoformulation techniques.

*Scutellaria baicalensis*, traditional Chinese medicine widely used in China, Japan, and other Asian countries, is clinically applied in various prescriptions, including Huanglian Jiedu Decoction, Gujing Pills, Puji Xiaodu Decoction, and Danggui Liuhuang Decoction. Aglycones in SBFC allow rapid absorption and good permeability, while glycosides show poor permeability and oral absorption. Glucuronidation and sulfation are the most essential metabolic pathways for SBFC administered orally. Glycosides participate in several cycles in the metabolic process to extend their retention time in the body, in which the liver is the main metabolic organ. The penetration ability across the BBB varies with the chemical structure of the drug administered but is usually quite limited or absent. The flavones in SBFC are metabolized rapidly and intensively in the small intestine and further in the liver, which is one of the reasons for their low bioavailability.

Currently, substantial progress has been made in administering SBFC in nanoformulations, especially in enhancing their solubility and bioavailability. Two remaining challenges in this field require further exploration. First, while much of the literature focuses on improving the bioavailability of nano-based techniques, very few studies provide a scientific interpretation or explanation as to how nanoformulations actually work to improve the bioavailability of SBFC. Second, while others describe important improvements or changes in ADME parameters, especially the metabolism and excretion profiles of SBFC in nanoformulations, few studies have evaluated further applications of nanopreparations. We hope that this review will capture the attention of researchers regarding nanoformulation, focus their studies on these issues, and provide useful pointers for future research.

## Figures and Tables

**Table 1:** Summary of the liposome components of *Scutellaria baicalensis.*

**Composition**	**Liposomal Carrier**	**Method of ** **Preparation**	**Outcomes**	**References**
Baicalin	Phospholipid	Reverse evaporation	PS 160-190 nm, the middle cerebral artery occlusion rats got higher *C*max and AUC0-t, which were about 1.5-2 times to normal rats both in BG and liposome groups.	[64]
Baicalin	Soybean lecithin	Reverse evaporation	After intranasal administration, compared with the BG group, the concentrations of t_1/2_, AUC0-t and BG in the brain tissue were significantly increased.	[26]
Baicalin	Phospholipon 90H	Effervescent dispersion technique	PS 373±15.5 nm, compared with BG suspension, the oral bioavailability was increased by 2.82 times, and the drug concentrations in liver, kidney and lung were increased by 5.39, 2.23 and 1.25 times, respectively.	[65]
Baicalin	Phospholipon 90H	Effervescent dispersion technique	PS 131.7±11.7 nm, administered intravenously in rabbits, concentration in the lung was the highest and significantly higher than that in the solution at each time point. the relative uptake rate and lung peak concentration ratio of targeting parameters were 4.837 and 2.789, respectively.	[66]
Wogonin	Soybean lecithin	Reverse evaporation	PS 90.5±2.2 nm, in the modified group, the accumulation rate and retention time in the liver were long, and the tumor inhibition rate was high.	[67]
Baicalein	Soybean phosphatidylcholine	Diethyl ether injection method	PS 709 nm, the bioavailability was 4.52 times that of BE solution.	[68]

**Abbreviations:** PS, particle size; AUC, area under the concentration-time curve; *C*max, maximum plasma concentration; t_1/2_, elimination half life

**Table 2:** Summary of nanoemulsions and SMEDDS of the components of *Scutellaria baicalensis.*

**Type of ** **Formulation**	**Composition**	**Method of ** **Preparation**	**Outcomes**	**References**
Nanoemulsions	Baicalin	Pseudo-ternary phase diagrams	PS BG-1 91.2 ± 2.36 nm BG-2 91.2 ± 2.36 nm, the AUC of BG-1 is 1.8-fold and 7-fold higher than that of BG-2 and the BG suspension.	[81]
	Baicalin	Pseudo-ternary phase diagrams	PS 58.43 nm, compared with BG suspension, the exposure is increased by 14.56 times. The intestinal absorption and lymphatic transport process contribute to its systemic exposure.	[82]
	Baicalin	Pseudo-ternary phase diagrams	PS 66.95 ± 17.32 nm, AUC and *C*max increased by 10.5-fold and 3.12-fold, respectively, compared with BG suspensions. In the rat lymph nodes treated with nanoemulsion, the *C*max increased 11.5 times.	[50]
	Baicalein	Emulsifying and high pressure homogenization	PS 90 nm, compared with suspension and conventional emulsion, the bioavailability increased by 524.7% and 242.1%, respectively.	[83]
SMEDDS	Baicalin	Water titration method	PS 228 nm, the results of single-pass intestinal perfusion and pharmacokinetics showed that SMEDDS could significantly promote the penetration of BG in the field, with a relative bioavailability of 220.3 ±49.93% and sustained release.	[87]
	Baicalein	Pseudo-ternary phase diagrams	PS 27.54 ± 9.59 nm, the *in vivo* results showed that the absorption of BE from SMEDDS resulted in about 200.7% increase in relative bioavailability compared with that of the BE suspension.	[88]
	Baicalein	Homogenized	PS 9.6 ± 0.2 nm, the relative bioavailability of SMEDDS and pc-SMEDDS were 342.5% and 448.7% respectively, which increased the lymphatic transport rate of BE from 18.8% to 56.2% and 70.2%, respectively.	[89]

**Abbreviations:** PS, particle size; AUC, area under the concentration-time curve; *C*max, maximum plasma concentration.

**Table 3:** Summary of other nanoformulations of the components of *Scutellaria baicalensis.*

**Type of ** **Formulation**	**Composition**	**Method of ** **Preparation**	**Outcomes**	**References**
Micelles	Baicalin	Solvent evaporation method	Improved absorption and circulation time in the intestine, *C*max and AUC are 1.77-fold and 1.54-fold of BG suspension.	[93]
	Baicalin	Thin film hydration method	PS 18 ± 0.5 nm, the distribution in the acute myocardial ischemia model is liver > heart > spleen > kidney > brain. The concentration of the drug in the heart shows an increasing trend, reaching 2897 ± 135 ng/mL at 120 min.	[94]
Nanostructured lipid carriers	Baicalin	The emulsion evaporation and low temperature solidification method	PS 83.9 nm, compared with BG solution, AUC increased by 7.2-fold and showed higher cardiac drug concentration.	[96]
	Baicalin	Emulsification and solvent evaporation method	PS 14-30 nm, the OX26 modification group can significantly increase the BG content in the brain, and SalB can be detected within 4 hours, which can promote the transmission of SalB and BG in the brain.	[97]
	Baicalein	Emulsification and ultrasound	PS 100 nm, in the brain distribution experiment, the accumulation of BE in cerebral cortex and brainstem was 7.5-fold and 4.7-fold higher than that in aqueous solution group, and increased about 2-3 times in the hippocampus, striatum, thalamus and olfactory tract.	[98]
Hydrogels	Baicalin	Welling-loading method	It has no obvious stimulation to the cornea and can prolong the anterior corneal retention time.	[100]
Solid dispersions	Baicalin	Solid dispersion technology	The pharmacokinetics of the BG co-precipitate capsules and the BG API capsules indicated that the mean values of *C*max were 127.04 ± 10.6 and 27.49 ± 36 μg/L, and those of AUC(0-24 h) were 1,080.23 ± 336.43 and 3,37.84 ± 127.64 μg/l × h, respectively. Compared with the BG API capsules, the relative bioavailability of the BG co-precipitate capsules was 338.2% ± 93.2%.	[102]
	Baicalin	Solvent evaporation method	AUC is 1.83-fold of pure BG, with shorter *T*max and higher *C*max, and no intestinal or renal toxicity.	[103]

**Abbreviations:** PS, particle size; AUC, area under the concentration-time curve; *C*max, maximum plasma concentration; *T*max, time to reach maximum concentration

## LIST OF ABBREVIATIONS

ADME: Absorption, Distribution, Metabolism, and Excretion
AUC: Area Under the Curve
BBB: Blood-brain Barrier
BCRP: Breast Cancer Resistance Protein
BCS: Biopharmaceutics Classification System
MRP: Multidrug-resistant Proteins
SB: *Scutellaria Baicalensis*
SBFC: SB Flavonoid Components
SLNs: Solid Lipid Nanoparticles
